# The impact of disasters and terrorism on the stock market

**DOI:** 10.4102/jamba.v11i1.534

**Published:** 2019-01-21

**Authors:** Tchai Tavor, Sharon Teitler-Regev

**Affiliations:** 1 Department of Economics and Management, the Max Stern Yezreel Valley Academic College, Israel

## Abstract

The growing number of negative events worldwide, among them natural disasters, artificial disasters and terrorism, has led the public to focus attention on the impact of such events on the economy and the capital market. This research examines the effects of natural disasters, artificial disasters and terrorism on the stock market in order to reveal profit opportunities. In this research, we collected data on 344 significant events that received media attention and examined the differences between the three types of events using the Pessimism Index. Some of the results include the following: (1) natural disasters cause the greatest damage to the economy, whereas terrorism causes the least damage; (2) natural disasters exhibit the highest level of severity, whereas artificial disasters have the lowest severity. The research reveals some opportunities for investors to obtain arbitrage profits. During natural disasters, the stock index decreases on the day of the events and on the two subsequent days. Therefore, investors should short sell the index on the day of the disaster and hold it for 2 days. On the contrary, during artificial disasters or terrorist incidents, the index drops only on the day of the event and the next day, so investors should short sell the index on the day of the disaster and hold it until the end of the first working day following the incident.

## Introduction

The growing number of natural disasters, artificial disasters^1^ and terrorist incidents around the world has led the public to focus attention on the impact of these events on various economic activities and on the growth of the global economy. These disasters and terrorist incidents, which are usually sudden and unpredictable, have increasingly affected a large portion of the world population over the last decades. Human activity enhances the effect of such events in that, as the population grows and building expands, previously unpopulated areas become more vulnerable.

Natural disasters are powerful natural phenomena that are caused by nature and that sow death and destruction in their wake. These natural events are marked by several criteria:

- They are caused by natural forces, but human actions can intensify or expedite them.
- The events are very powerful and are measured by specified indices, such as the Richter scale for measuring the magnitude of earthquakes or the Volcanic Explosivity Index for measuring the relative explosiveness of volcanic eruptions.
- The events are very sudden, last a short time and are unpredictable (or give very short notice as in the case of a tsunami).
- The events cause casualties and major economic damage.

Countries around the world are vulnerable to different natural hazards. In some countries, earthquakes and volcanic eruptions occur frequently; in others, typhoons and landslides are common, while still others suffer frequent floods or droughts. Nevertheless, in examining the ways and abilities to cope with natural disasters, it is important to distinguish between developed countries that invest major sums of money in dealing with the hazards of nature and developing countries that have no budget for coping with such hazards.

The cost of natural disasters has increased rapidly in recent years, reaching over a 100 billion dollars annually (National Center for Environmental Information n.d.). One of the largest natural disasters was the tsunami in Indonesia in 2004. The earthquake in the Indian Ocean, measuring 9.3 on the Richter scale, was one of the deadliest in modern times, and it created huge tsunami waves that washed over the northwest part of Indonesia. Over 230 000 people were killed by the tsunami, more than 1 million people were left homeless and the economic damage exceeded $3bn (Encyclopedia Britannica n.d.:1 of 2).

The term ‘disaster’ is used to describe an event or a situation that sows destruction, affecting life, health, property and the surroundings. Disasters are usually caused by human beings and can include airplane crashes, train accidents and building collapses, just to name a few. The numbers of people killed in such disasters are usually small compared to natural disasters, but the economic damage can be larger. One of the biggest disasters of the last decade was the collision of a passenger train and a freight train in Los Angeles in 2008, resulting in the deaths of 25 passengers and economic damage of over $500m (Rubin, Simmons & Landsberg 2008).

Terrorism is an act of violence directed towards civilians. Terrorism strives to achieve political, religious or social goals using fear. In recent years, terrorists changed and developed their method and usually hit organisations in open economic channels (Abadie & Gardeazabal 2008:20).

Conventional terrorism has limited ability to do damage that is able to convey the terrorist message to an audience different from that of the organisation and first and foremost to the population of the affected country. Yet, some major terrorist incidents have caused major damage. The two major events in the last decade are as follows (Encyclopedia Britannica n.d.):

- The attack of 11 September 2001, which took the lives of 3000 people, caused 430 000 employees to lose their jobs and resulted in economic damage of over $30bn (https://www.history.com/topics/9-11-attacks).
- The train terror attacks in Madrid in 2004 that resulted in 192 deaths, 1500 injured and billions of dollars in economic damage (p. 2 of 2).

The purpose of this research is to compare the effect of different types of events on the capital market.

### Literature review

Many researchers have studied the impact of natural disasters on the economy. They found that such disasters have a significant negative effect on the real gross domestic product (GDP) in the affected countries and that major events affect both short-run and long-run output while more minor events affect neither long-run nor short-run output. They also found that climate disasters encourage the accumulation of human capital and technological development, which in turn effect economic growth, whereas geological disasters lead to the destruction of human capital (Bergholt & Lujala 2012:20; Cavallo et al. 2013:34; Cuaresma, Hlouskova & Obersteiner 2008; Kim 2011:19; Skidmore & Toya 2002:682).

Focusing on the effect of natural disasters on the financial market, Noy (2009:229) found that higher per capita income and greater openness to trade as well as more government spending increase the market’s ability to avoid the effects of a natural disaster on the country’s economy. In addition, higher foreign currency reserves and higher levels of domestic credit together with capital accounts that are less open appear more robust and better able to endure natural disasters. Studying the capital market in Australia, Worthington and Valadkhani (2004:16) used data from 31 December 1982 through 01 January 2002 and found that bushfires, cyclones and earthquakes had a major effect on market returns, whereas severe storms and floods did not have a similar effect. The net effects can be positive and/or negative. Most of the effects were felt on the day of the event, with some adjustment on the days that followed.

In a later study, Worthington (2008:2185) used daily returns on the Australian capital market between 1980 and 2003 to study the effect of natural events and disasters. He found that the disasters were not responsible for any of the volatility in stock value.

Some researchers have studied the effect of aviation disasters on the capital market. Bosch, Eckard and Singal (1998:515) examined whether there is a spillover effect or a switching effect on rival companies. They found a switching effect when the non-crash airline overlapped with the crash airline and a spillover effect when there was no such overlapping. Kaplanski and Levy (2010:178, 196), for example, used data from January 1950 to December 2007 to examine the New York Stock Exchange (NYSE) rate of return. The researchers assumed that aviation disasters affect people’s mood and anxiety, which in turn have a negative effect on the capital market. The model they used included the stock index return as an independent variable and a dependent dummy variable representing the day of the week, the day after a holiday, the first days in the tax year and the size of the effect. The event window began on the day before the event and continued three days after the event. The researchers found a significant effect, with an average market loss of more than $60bn per disaster, although the actual loss was less than $1bn. On the third day after the disaster, the stock prices started to increase. They also found that the effect was larger when the stock was smaller and riskier and when the stock belonged to a less stable industry.

Capelle-Blancard and Laguna (2010:204) studied the effect of industrial disasters on the capital market using companies related to the accident and not the stock index market. They used data covering the years 1990 through 2005 and found that the capital market was negatively affected by an accident in the 2-day period after the event. The effect was strongly related to the severity of the event and to the level of the company’s safety records. Focusing on a single disaster with long-lasting effects in which the damage increased during the five months after the event, Kollias, Papadamou and Stagiannis (2012:197) researched the effect of a deep-water horizon accident – the 2010 Gulf of Mexico oil spill – on the stock values of firms in the gas and oil industry. As might be expected, the results showed a decrease in the stock value of the involved company and indeed of the entire industry.

Terrorism is a form of violent struggle usually directed against civilians. The purpose of terrorism is to create fear in an attempt to achieve political, religious or social goals. During recent years, terrorist groups have made significant advances in their methods and in the technology they use to carry out terrorist incidents. The organisations hit hardest by terrorism are the open economic channels that allow unlimited movement of people, capital, products and service among countries around the world (Abadie & Gardeazabal 2008:20).

The effect of terrorism on the economy and on financial markets has been thoroughly researched by several researchers. Their findings show that in countries where foreign trade, tourism and foreign investments are more important than other financial sectors, the aggregate financial loss will be greater, and that diversity is the most important element in the solvency and stability of financial markets although a flexible reaction on the part of authorities is also very important (Harrigan & Martin 2002:107; Johnston & Nedelescu 2006:23; Sandler & Walter 2004:314).

Eldor and Melnick (2004:385) used data for the period 1990 through 2003 on the Israeli stock and bond market to study the effect of 639 terrorist incidents. They used several parameters, including the location and target of the incident, the type of terrorist incident and the number of casualties and wounded. The results showed a negative and permanent effect on the capital market since the beginning of the Al-Aqsa Intifada in September 2000. In addition, suicide bombings and bombings in which victims were wounded had a permanent negative effect on the market, whereas attacks on transportation and attacks within the Green Line had a transitory negative effect on the market. On the contrary, looking at the same time period in Israel, Peleg et al. (2011:278) showed that although Israel’s financial base remained sensitive to each act of terror, sustained psychological resilience was indicated with no apparent overall market shift.

Researching the same issue, Tavor (2011:79) found that the response of the capital market is larger in non-confrontational areas and in central areas. Brounen and Derwall (2010:597) researched the effect of 31 terrorist incidents in the period 1991–2005 on eight important financial markets. They found an average drop of 0.34% on all the markets during the day of the event and a drop of 0.92% on the market where the terrorist activity took place. In addition, they found that the market returned to its original values in 2–3 days. A similar result was achieved by Nikkinen and Vahamaa (2010:273), who examined the impact of terrorist activities on the British capital market and found a large drop on the Financial Times Stock Exchange (FTSE) index in the short-term after a terrorist attack. Bilson et al. (2012:57) focused on major terrorist incidents (more than 10 casualties and significant economic damage) and found that terrorist incidents cause significant ‘infection’, mainly in developed countries, but that the average effect lasts only for a short period of time.

Chesney, Reshetar and Karaman (2011:266) tested the impact of terrorist incidents on the capital market, the bond market and the commodity market in several countries. They found that two-thirds of the terrorist incidents had a strong and significant effect on all markets. Industrial companies and airlines were most sensitive, whereas banking was less sensitive. Terror has a negative effect on gold and other commodities as well. The researchers found that the effect is strongest on the day after the event and declines on the following days. They also compared the effect of terror to the effect of other disasters and found several similarities. In the case of natural disasters, however, the negative impact is usually observed in the post-event period, meaning that markets need more time to evaluate the long-term effect of such events.

In this article, we attempt to bridge the gap in the literature by comparing the effect of different events on the capital market. Currently existing research focuses on terrorist incidents and on artificial or natural disasters and their effect on the capital market, without taking into consideration the type of event (natural disaster, artificial disaster or terrorism). Measuring and comparing the impact of different types of events can help in understanding the way people evaluate different types of risk.

In addition, most previous research focused on the effect of a particular type of event, that is, air crash or earthquake, and on a certain industry, that is, airline or company. This research, in contrast, focuses on the effect of a disaster on the stock value in the country where the disaster occurred.

## Research method and design

### Data

In this research, we collected data for three types of events: artificial disasters, natural disasters and terror incidents that took place in different countries around the world from 02 September 1983 through 06 March 2013. Data were collected from several websites that cover international disasters and included the following: a description of the event, the number of casualties and fatalities, the location of the event, the estimated financial damage and other data regarding the country where the event occurred. The sample includes 344 significant events that received media attention: 127 artificial disasters, 88 natural disasters and 129 terrorist incidents. Data regarding each country’s major financial indices were collected from YahooFinance.com, Investing.com and Bloomberg.com, which serves as the main source of information regarding the major capital market indices around the world.

### Methodology

Three types of events affect the economy and the capital market in a particular country. In this research, the focus was on the effect of particular events on the capital market and on whether the effect of different types of events was similar or whether each type of event had a different effect. Data were collected for each event on the day before the event and for the 30 days following the event. Nevertheless, because the events we examined are heterogeneous and because natural disasters typically have a long-lasting effect while terror incidents are short-term, the research focused only on the 2 days following the event in order to create a common ground for comparison. To this end, the study examined the closing prices of the central index in the targeted country on the day of the incident (*Y*_t_), on the day before the incident (*Y*_t-1_) and on the 2 days following the incident (*Y*_*_t_*__+1_ and *Y*_*_t_*__+2_).^2^ If the event took place during the trading day or in the morning of the trading day, the trading day was counted as the day of event. If the event took place after the close of the trading day or on a day without trade, the first trading day after the event was considered the day of the event.

In order to test the effect of the event, we used the Pessimism Index (PI), which assesses pessimism on a scale ranging from 4 to 13 (as discussed subsequently). The PI is commonly used in investigating terrorist incidents (see Araz-Takay, Peren & Omay 2009:3; Eckstein & Tsiddon 2004:988; Eldor et al. 2008:9, 2012:21), although in a slightly different way. We adjusted the PI to be able to test other types of events. Although the model in previous studies used the Terror Index (TI) around the day of the event, this research uses the return around the day of the event and the PI index on the day of the event. We examined only 2 days after the event, based on previous research that found that the effect was short-term and that after the second day stock prices rose again (see Kaplanski & Levy 2010:196; Worthington & Valadkhani 2004:16).^3^

The econometric model in this research is represented by Equations 1 to 3:

$$Y_{\text{t}}=\text{α+}\beta_{1}\cdot Y_{\text{t}-1}+\text{γ}\cdot PI_{\text{t}}+\varepsilon_{\text{t}}$$ [Eqn 1]

$$Y_{\text{t+1}}=\text{α+}\beta_{1}\cdot Y_{\text{t}-1}+\beta_{2}\cdot Y_{\text{t}}\text{+γ}\cdot PI_{\text{t}}+{\text{ε}}_{\text{t}}$$ [Eqn 2]

$$Y_{\text{t+2}}=\text{α+}\beta_{1}\cdot Y_{\text{t}-1}+\beta_{2}\cdot Y_{\text{t}}\text{+}\beta_{3}\cdot Y_{\text{t}+1}\text{+γ}\cdot PI_{\text{t}}+{\text{ε}}_{\text{t}},$$ [Eqn 3]

where *Y*_*_t_*_ represents the return of the central index on the day of the event, *Y*_t-1_ represents the return of the index a day before the event, *Y*_t+1_ represents the return of the central index on the day after the event and *Y*_t+2_ represents the return of the central index 2 days after the event.

The Pessimism Index has been used extensively in the past in investigating terrorist incidents. This index reflects the level of investors’ pessimism in the capital market following a particular event. We extended the index to all types of events. In building the index, four different parameters involved in deciding on the intensity of the event were considered.^4^ The first parameter is the number of fatalities in the event (D_1_). For this parameter, four categories ranging from 1 to 4 were defined, where 1 represents an event with up to 100 fatalities, 2 represents an event with 101–1000 fatalities, 3 represents an event with 1001–10 000 fatalities and 4 represents an event with more than 10 000 fatalities. The second parameter is the number of casualties (D_2_), with four categories similar to those for the number of fatalities. The third parameter (D_3_) is the location of the event. This parameter has two categories: 1 represents an event in a non-central area or city, and 2 represents an event in a major city or central area. D_4_ represents the estimated damage of the event. This parameter has three categories: 1 represents economic damage less than $100m, 2 represents economic damage between $100m and $1bn, and 3 represents economic damage greater than $1bn. The PI event severity index is based on these parameters. For example, the PI index for the September 11 terror attack is: 12 (the number of deaths was 2996, therefore D_1_ = 3; the number of casualties was 14 837, therefore D_2_ = 4; the attacks happened in major cities, therefore D_3_ = 2; and the total damage was over $1bn, therefore D_4_ = 3).

$$PI=D_{1}+D_{2}+D_{3}+D_{4}$$ [Eqn 4]

The level of the index ranges from 4 to 13. A higher value on this index indicates an event that caused a great deal of damage in a central location, whereas an index level of 4 indicates an event with little damage in a non-central location.

**Hypothesis 1:** All types of event will influence the expected profitability of companies and therefore the risk premium because of the increased level of uncertainty, which causes a decline in the share prices. The main hypothesis is that there will be negative correlations between the events and the capital market and that these correlations will be more negative if the severity of the event is greater.

This hypothesis is in line with previous studies; among them are Worthington and Valadkhani (2004:16) and their research on natural disaster, Kaplanski and Levy (2010:196) on aviation disasters, Capelle-Blancard and Laguna (2010:204) on industrial disasters, and Brounen and Derwall (2010:597), Nikkinen and Vahamaa (2010:273) and Tavor (2011:79) on terror.

**Hypothesis 2:** The effect of different disasters on the capital market will vary based on the type of event. Chesney et al. (2011:266) found that it takes the financial market longer to react to natural disasters.

## Results

### Descriptive statistics

This section provides a general description of the data that were collected for this research and their impact on the capital market, as shown in Table 1.

**TABLE 1:** Statistical data on event severity and type.

Panel	Location	*N*	Mean	Median	SD	Min.	Max.
Panel A: Artificial disasters	Fatalities	127	0.95	1	0.73	1	3
	Casualties	127	0.64	1	0.70	1	3
	Central city or area	127	1.49	1	0.50	1	2
	Financial loss	127	1.59	2	0.54	1	3
	*PI*	127	4.65	5	1.31	4	11
Panel B: Natural disasters	Fatalities	88	1.49	1	1.18	1	4
	Casualties	88	2.01	2	1.34	1	4
	Central city or area	88	1.51	2	0.50	1	2
	Financial loss	88	2.44	3	0.73	1	3
	*PI*	88	7.34	7	3.00	4	13
Panel C: Terror incidents	Fatalities	129	1.02	1	0.49	1	3
	Casualties	129	1.26	1	0.68	1	4
	Central city or area	129	1.62	2	0.49	1	2
	Financial loss	129	1.16	1	0.44	1	3
	*PI*	129	5.05	5	1.35	4	12

*N*, number; SD, standard deviation; Min., minimum; Max., maximum; *PI*, panel index.

Table 1 depicts the PI and its components based on type of event. The table includes three panels: panel A represents artificial disasters, panel B represents natural disasters and panel C represents terrorist incidents. The table shows that the number of fatalities and casualties is higher for natural disasters (M*Fatalities* =1.49 and M*Casualties* = 2.01, respectively), while lower levels of fatalities and casualties are associated with artificial events (M*Fatalities* = 0.95 and M*Casualties* = 0.64, respectively). Data for the location of the event show that 62% of the terrorist incidents took place in central locations (M*Central* = 1.62), whereas only 50% of natural and artificial disasters took place in central locations (M*Central* = 1.49 and M*Central =* 1.51, respectively). This is because the purpose of terrorism is to create fear and horror and to attract media attention, and therefore, terrorists target central locations. On the contrary, artificial and natural disasters are usually unintentional, so that the probability they will occur in a central or a non-central location is similar.

The economic damage caused by these three types of events is large and influences not only the affected area but the country’s economy as a whole. Table 1 shows that the economic damage is highest in natural disasters (M*Financial_loss* = 2.44) and lowest in terrorism (M*Financial_loss =* 1.16).^5^ An evaluation of the severity of the events shows that the severity of natural disasters is highest (*PI* = 7.34), whereas the severity of artificial disasters is lowest (*PI* = 4.65).

Table 2 depicts the return performances of the main indices in the relevant countries over the course of the 4 days around the event (*t* = -1, +1, +2). The table has three panels: Panel A represents artificial disasters, panel B represents natural disasters and panel C represents terrorist incidents. The table shows that on the day before the event there is no difference between the three types of events and the returns on the indices do not vary. On the day of the event, all three types show negative returns, with the most negative returns for terrorist incidents, *M*_*terror*_, *Y*_*t*_ = -1.459%, which usually show panic and fear among the local population. The least negative return is for artificial events, when *M*_*artificial*_, *Y*_*t*_ = -0.693%. To test whether these negative returns continue after the event, the researchers tested the returns of the indices during the 2 days following the events.^6^

**TABLE 2:** Influence of type of event on the capital market during the 4 days around the event.

Panel	Indices	*N*	Mean (%)	Median (%)	SD (%)	Percent negative (%)	Min (%)	Max (%)
Panel A: Artificial disasters	*Y*_*t-1*_	127	−0.001	0.000	0.302	44.3	−6.868	4.464
	*Y*_*t*_	127	−0.693	−0.288	0.379	53.6	−6.545	11.279
	*Y*_*t+1*_	127	−1.146	−0.436	0.430	68.4	−16.511	6.294
	*Y*_*t+2*_	127	−0.351	0.000	0.438	49.7	−11.571	7.365
Panel B: Natural disasters	*Y*_*t-1*_	88	−0.026	−0.022	0.206	42.8	−4.922	4.902
	*Y*_*t*_	88	−1.049	−0.574	0.364	64.8	−10.571	6.553
	*Y*_*t+1*_	88	−1.641	−0.643	0.377	73.9	−10.554	1.559
	*Y*_*t+2*_	88	−1.141	−0.435	0.384	71.3	−7.916	5.678
Panel C: Terror incidents	*Y*_*t-1*_	129	0.107	0.194	0.222	47.6	−6.114	3.009
	*Y*_*t*_	129	−1.459	−0.548	0.394	75.4	−10.357	5.500
	*Y*_*v+1*_	129	−0.975	−0.490	0.363	59.1	−7.730	4.450
	*Y*_*t+2*_	129	−0.037	0.000	0.255	44.6	−7.558	4.436

*N*, number; SD, standard deviation; Min., minimum; Max., maximum; *Y*_*_t_*_, Indices on Day t.

The results show that natural disasters had the highest negative effect on the capital market, whereas the index returns show an average decrease of 2.782% during the 2 days after the event. The other two types of events show a negative effect that lasts only 1 day after the event, with an average decline of 1.146% for artificial disasters and of 0.975% for terrorist incidents. This table and Table 1 support Hypothesis 2, which stated that different types of events will have a differential effect on the stock market. The results in the tables show that natural disasters are the most severe and therefore have a greater impact on the capital market.

### Regression estimates

We then used linear regression to test the correlations among the daily returns in the event window (-1, 0, +1, +2). We tested whether the daily return on each of the days since the event correlates with the return on the days prior to the event. In addition, we tested the correlation between the return of the indices on the day of the event and the severity of the event. Table 3 depicts the econometric model for this test for Equations 1–3. The table includes three panels: panel A represents artificial disasters, panel B represents natural disasters and panel C represents terrorist incidents.

**TABLE 3:** Regression estimates comparing the effect of three types of events – artificial disasters (Eqn 4), natural disasters (Eqn 5) and terrorist incidents (Eqn 6).

Variable	Panel A: Artificial disasters	Panel B: Natural disasters	Panel C: Terrorist incidents
**Equation 4**			
C	−0.004 (0.007)	−0.003 (0.006)	−0.005 (0.008)
*Y_t-1_*	−0.122 (0.096)	−0.132 (0.161)	0.160 (0.141)
PI	0.037 (0.073)	0.004* (0.002)	−0.002 (0.002)
**Equation 5**			
C	−0.004 (0.008)	−0.003 (0.006)	−0.006 (0.009)
*Y*_*t-1*_	−0.135 (0.103)	−0.138 (0.177)	0.179 (0.151)
*Y_t_*	0.069* (0.026)	0.126* (0.041)	0.222* (0.093)
PI	0.041 (0.079)	0.004* (0.002)	−0.002 (0.002)
**Equation 6**			
C	−0.005 (0.008)	−0.003 (0.007)	−0.007 (0.009)
*Y*_*t-1*_	−0.150 (0.112)	−0.145 (0.193)	0.199 (0.161)
*Y*_*t*_	−0.122 (0.084)	0.009 (0.006)	−0.208 (0.142)
*Y*_*t+1*_	0.076* (0.029)	0.131* (0.045)	0.246* (0.099)
PI	0.045 (0.085)	0.005* (0.002)	−0.001 (0.002)

SD, standard deviation; Eqn, equation; PI, panel index, C, constant.

* , *p* < 0.05.

This table tests the correlation between event severity and index returns on the 4 days surrounding the event (*t* = -1, 0, +1, +2).

Regression estimates comparing the effect of three types of events – artificial disasters, natural disasters and terrorist incidents – on the capital market are provided in Table 3.

The econometric model exhibits in Equations 5 to 7.

$$Y_{\text{t}}=\alpha +\beta_{1}\cdot Y_{t-1}+\text{γ}\cdot PI_{\text{t}}+\varepsilon_{\text{t}}$$ [Eqn 5]

$$Y_{\text{t+1}}=\alpha +\beta_{1}\cdot Y_{\text{t}-1}+\beta_{2}\cdot Y_{\text{t}}\text{+γ}\cdot PI_{\text{t}}+\varepsilon_{\text{t}}$$ [Eqn 6]

$$Y_{\text{t+2}}=\alpha +\beta_{1}\cdot Y_{\text{t}-1}+\beta_{2}\cdot Y_{\text{t}}\text{+}\beta_{3}\cdot Y_{\text{t+1}}\text{+γ}\cdot PI_{\text{t}}+\varepsilon_{\text{t}}$$ [Eqn 7]

The results in Table 3 show that there is no correlation between the index returns on the day of the event and the returns on the day before the event for all three types of events. The results for artificial disasters and terror events are similar, meaning there is a linear correlation between the index returns on the day of the event and their returns after the event, as can be seen from Equation 5. This means that if there is a decline in the index on the day of the event, investors have the opportunity to build a profitable strategy and short sell the index on the day after the event. In addition, there is no correlation between the index returns on the day of artificial disasters or terrorist incidents and the returns 2 days after the event, indicating that the effect of the event is short-term (Equation 6). The severity of the event does not affect the performances of the indices on the day of the event. This is in line with the results of Worthington and Valadkhani (2004:16).

In examining natural disasters, we found that they have a different effect on the capital market. For this type of event, there is a correlation between the index returns on the day of the event and the returns on the 2 days following the event. This means that if there is a decline in the index on the day of the event, investors can build a profitable strategy and short sell the indices on the 2 days following the event. With respect to event severity, we found a correlation between severity and index returns on the day of the event. This means that a higher PI points to more negative returns from the event. In conclusion we can say that natural hazards have a stronger impact on the capital market.

## Discussion

This research tested two hypotheses. Hypothesis 1 assumed that there would be a negative correlation between the events and the capital market and that this correlation would be more negative if the event was more severe. The results of the analytical model fully support this hypothesis, in line with previous research (Brounen & Derwall 2010:597; Capelle-Blancard & Laguna 2010:204; Kaplanski & Levy 2010:196; Nikkinen & Vahamaa 2010:273; Tavor 2011:79; Worthington & Valadkhani 2004:16).

Hypothesis 2 was also confirmed because we found that the impact of a natural disaster lasts longer and that events of that type have a larger effect, in line with previous research (Chesney et al. 2011:266).

### Implications

The results indicate that all three types of events showed an average decline in the indices on the day of the events and on the days that follow. On the day of the event, the strongest decline is for terrorist incidents and the smallest decline is for artificial events. In the case of event severity, the largest decline exhibited for natural disasters and not for terrorist incidents.

A possible explanation for the larger decline on the day of a terrorist incident than on the day of a natural disaster might be a psychosocial one. Many terrorist organisations strive to wage a moral-psychological battle against the population by sending threatening messages and posting videos prior to the incident. Such activities cause more fear and uncertainty among investors, resulting in a larger decline of the stock indices. Natural disasters provide no such prior announcements, and therefore, it takes investors longer to translate the event into a decline on the indices, despite the event’s severity.

Based on this research, further research is recommended to test the influence of disasters on the stock market based on the characteristics of the different countries, such as whether a country is developed or developing, a democracy or a dictatorship, among others.

### Limitations

This study has two limitations. One limitation is the difficulty in gathering historical data from countries that experienced major disasters affecting their economy and their capital market.

The other limitation is the problem in determining investment strategies from intra-daily data at the day of the event. Some of the events happened in countries where intra-daily data were not available after the fact. Therefore in this research, we used daily data.

## Conclusion

Over the last century, many countries have experienced both natural and artificial disasters, as well as terrorist incidents. These events have affected not only the area or the people involved but also the nation’s economy.

The main focus of this research was to examine the effects on the capital market of three types of events: natural disasters, artificial disasters and terrorism. The research analyses the effects of event severity on the capital market returns.

The descriptive statistics show that natural disasters cause the greatest economic damage, whereas terrorism causes the least damage. In addition, the number of casualties and deaths is highest for natural disasters and lowest for artificial disasters, so that the severity of natural disasters is highest, whereas the severity of artificial disasters is lowest.

Therefore, our finding that natural disasters have the highest negative impact on the capital market and that this impact continues to decline during the 2 days after the event is a reasonable finding. The other two types of events have a negative effect that lasts only 1 day after the event.

Terrorist incidents usual occur in central cities because of the terrorists’ goal of attracting as much public attention as they can, a goal that can be more easily achieved in central cities. Artificial disasters and natural disasters are unpredictable and arbitrary and therefore distributed evenly among central and non-central locations.

The effects of natural or artificial disasters as well as of terrorist incidents on the economy of countries as reflected by the capital market are very important and are the focus of this research. It is important to see how different types of events affect the capital market and whether their effects vary.

The research results show that natural disasters have the highest negative impact on the capital market and that this effect lasts longer than the effect of the other two types of events, which lasted only 1 day after the event. This finding supports earlier results like those of Chesney et al. (2011:266) and may be explained by the fact that such events cause the greatest economic damage and the highest number of casualties and deaths.

The outcome of the regression model shows that there is no correlation between the index returns on the day of the event and the returns on the day before the event for all three types of events. Artificial disasters and terrorist incidents behave similarly, with both showing a linear correlation between the indexes return on the day of the event and the return after the event, indicating that the event’s effect is short-term. The severity of the event does not affect the performances of the indices on the day of the event.

Natural disasters have a different effect on the capital market. In this case, there is a correlation between the index return on the day of the event and the return on the 2 days following the event, indicating that the event has a longer effect (see also Chesney et al. 2011:266). There is a correlation between event severity and the index return on the day of the event, indicating that the higher the PI, the more negative the event return will be. In conclusion, we can say that natural disasters have a stronger effect on the capital market.

Both of the research hypotheses were corroborated. Specifically, Hypothesis 1 was fully corroborated in that we found a negative correlation between disaster events and the capital market and that the correlations are more negative if the event is more severe.

Regarding Hypothesis 2, we found, in line with previous research (Chesney et al. 2011:266), that the effect of a natural disaster lasts longer and that natural disasters have a larger effect.

The research reveals some opportunities for investors to make a profit. If they short sell the index on the day of such an event, they can gain some positive returns on the following days. Further research is recommended to compare the financial behaviour in developing versus developed countries, democratic versus non-democratic countries and so on.

This research tests whether investors have the option to build a strategy that on average will beat the stock market based on three types of events. The research focuses on characteristics of the event, such as the number of injured and dead, economic damage and the like. Of course, there are other factors that affect the stock market based on the characteristics of the given country, such as the size of the economy and its financial situation. Nevertheless, this research focused on the events themselves and not on the country in which they occurred. Further research may examine the effect of an event on the stock market based on the characteristics of the given country whether it is developed or developing country, small or big, and so on.

## Footnotes

1 The term ‘artificial disasters’ refers to disasters not caused by nature.

2 The time that was tested was longer, but most of the damage and uncertainty was on the 2 days following the event. Therefore, the research focused on this period.

3 Natural disasters can last longer than 2 days, and therefore, their effect may be longer lasting. This has been researched in the past. The purpose of this article is to compare the effect of different types of events. Therefore, the smallest common denominator of 2 days was used.

4 In order to check the robustness of the results, we divided the groups differently and received similar results.

5 If psychological effect and fear among the population could be easily measured in financial terms, the economic damage of terror incidents would likely be larger compared to the other two types of events.

6 We tested only 2 days after the events based on Nikkinen and Vahamaa (2010:273), who found that the effect of such events is short-term.
